# Why the same herb is not the same medicine: plant epigenetic and metabolic plasticity as a missing variable in Chinese medicine

**DOI:** 10.1186/s13020-026-01434-9

**Published:** 2026-05-29

**Authors:** Tae Kyung Hyun

**Affiliations:** Department of Industrial Plant Science and Technology, College of Agriculture, Life and Environment Sciences, Chungbuk National University, Cheongju, 28644 Republic of Korea

**Keywords:** Biological memory, Epigenetic regulation, Metabolic plasticity, Quality control

## Abstract

Traditional Chinese medicine (TCM) standardization is largely based on chemical equivalence, yet achieving reproducible therapeutic efficacy remains challenging. This limitation reflects an implicit assumption that medicinal plants are chemically static materials, overlooking their nature as dynamically regulated biological systems. This commentary emphasizes plant epigenetic and metabolic plasticity as a missing dimension in current quality assessment strategies. Environmental and developmental factors can establish stable regulatory states through epigenetic mechanisms, including DNA methylation and histone modification, thereby shaping metabolic organization beyond immediate growth conditions. Because therapeutic efficacy emerges from coordinated metabolic networks, evaluation based solely on static chemical composition is inherently limited. I propose an epigenetically informed materia medica framework for next-generation quality assessment. By integrating dynamic metabolic signatures, such as metabolite relationships and pathway coordination, with epigenetic indicators of regulatory history, this approach redefines consistency as predictable functional performance rather than chemical uniformity. This perspective provides a biologically realistic foundation for improving reproducibility while aligning standardization with the systemic principles of herbal therapeutics.

## Background

Medicinal plants constitute the intellectual and practical foundation of traditional Chinese medicine (TCM), in which therapeutic efficacy has long been attributed to coordinated interactions among multiple constituents rather than to isolated active principles. In parallel with the modernization of TCM research, considerable efforts have been devoted to improving botanical authentication, standardizing processing procedures, and strengthening chemical quality control through advanced analytical technologies [1–3]. These advances have substantially enhanced safety, traceability, and regulatory reliability. However, these approaches are largely grounded in the assumption that chemical equivalence ensures biological equivalence, an assumption that does not always hold for metabolically dynamic plant materials. As a result, achieving reproducible therapeutic efficacy across batches remains a persistent challenge in both experimental investigations and clinical practice.

This inconsistency highlights a fundamental limitation in the prevailing research premise. The premise assumes that once species identity and chemical composition are verified, medicinal plants can be regarded as relatively uniform materials. However, accumulating evidence in plant biology challenges this view. Research increasingly shows that plants function as dynamically regulated systems whose metabolic outputs are continuously shaped by environmental conditions, development stages, and regulatory states established during growth, rather than being determined solely by genotype [4]. Recognizing this complexity raises a critical yet understudied question for TCM research. To what extent does the intrinsic biological plasticity of medicinal plants contribute to variability in therapeutic efficacy beyond what can be explained by conventional quality parameters? Importantly, not all variability is equivalent. While uncontrolled variability arising from poor handling, contamination, or measurement error represents noise that should be minimized, a substantial portion reflects structured biological responses shaped by environmental and developmental regulation. The central challenge is therefore not variability per se, but the failure to distinguish and interpret biologically meaningful variation within appropriate regulatory contexts. Addressing this challenge requires a conceptual shift from static chemical descriptors toward a framework that understands medicinal herbs as environmentally programmed and biologically responsive materials.

In this commentary, I argue that plant epigenetic and metabolic plasticity constitutes a missing dimension in current interpretations of herbal consistency and efficacy. Incorporating this perspective is essential not only for deepening mechanistic understanding but also for reshaping standardization strategies in ways that are biologically realistic and consistent with the foundational principles of TCM.

### Metabolic plasticity of medicinal plants

Medicinal plants continuously adjust their metabolism in response to environmental and developmental conditions [4]. These external factors profoundly influence the production and allocation of secondary metabolites, reflecting adaptive metabolic programs rather than minor fluctuations. Accumulated evidence suggests that the biosynthesis of these therapeutically important compounds is tightly linked to stress responses and ecological interactions, resulting in substantial variation in composition and relative abundance [5]. Consequently, the metabolome of a medicinal plant is a dynamic, condition-dependent network, not a static chemical profile. This metabolic plasticity is not random, but is driven by coordinated regulatory mechanisms that reshape entire metabolic pathways and alter inter-metabolite relationships. From this perspective, variability in medicinal efficacy is an inherent consequence of plant metabolic flexibility, rather than an artifact of quality control. Crucially, such variability should not be interpreted as uncontrolled fluctuation but as structured variation arising from coordinated metabolic regulation. Distinguishing this biologically programmed variability from non-biological noise is essential for accurate quality assessment. Recognizing medicinal plants as metabolically plastic systems therefore provides a critical foundation for understanding why chemical equivalence does not always translate into functional equivalence.

For example, in *Artemisia annua*, artemisinin biosynthesis and accumulation are highly sensitive to environmental conditions [6]. Such variation directly influences the yield of this frontline antimalarial compound, thereby linking metabolic plasticity to therapeutic performance. More broadly, secondary metabolite accumulation in medicinal plants is shaped by environmental and developmental factors, which alter both the composition and relative abundance of bioactive compounds [7]. These responses reflect coordinated regulation of biosynthetic pathways rather than isolated changes. Collectively, these observations support the view that the plant metabolome is a condition-dependent and dynamically regulated system, underscoring the limitations of static chemical equivalence as a predictor of therapeutic efficacy.

### Epigenetic contributions to metabolic plasticity in medicinal plants

Metabolic plasticity in medicinal plants raises an important mechanistic question about how environmentally induced metabolic states are stabilized and maintained over time. Epigenetic regulation provides a compelling explanation for this continuity. In plants, DNA methylation, histone modification, and chromatin remodeling act as central mechanisms that integrate environmental signals into durable transcriptional programs [8]. These processes allow plants to convert transient stimuli into longer-lasting regulatory states. Emerging evidence suggests that environmentally induced metabolic variation can be stabilized through epigenetic mechanisms. In medicinal plant systems, stress exposure and cultivation history have been linked to persistent changes in DNA methylation and chromatin state at loci associated with secondary metabolism, leading to sustained alterations in metabolite profiles [8]. For example, studies in *Glycyrrhiza uralensis* and related species show that environmental conditions modulate secondary metabolite biosynthesis in a manner consistent with epigenetically mediated transcriptional regulation [9, 10]. These observations support the view that metabolically relevant states underlying medicinal quality reflect not only current environmental conditions but also the regulatory memory of prior exposure, and that such epigenetically mediated metabolic shifts can ultimately influence bioactivity-relevant properties of medicinal plants. This provides a direct link between epigenetic state, metabolic reprogramming, and functional outcomes relevant to therapeutic efficacy. Importantly, epigenetic regulation does not operate in an on–off manner but fine-tunes gene expression across entire metabolic networks. Genes involved in secondary metabolism are particularly sensitive to chromatin state, which enables coordinated regulation of pathway activity rather than isolated control of individual enzymes [8, 11]. Exposure to specific biological and environmental contexts can therefore establish epigenetic configurations that bias metabolic fluxes long after the original stimulus has subsided. Through this mechanism, environmental information is integrated into stable regulatory programs that shape metabolic behavior over time. This mechanistic insight has important implications for how medicinal plant quality is currently assessed. Contemporary approaches to standardization are predominantly grounded in static chemical evaluation. The quantification of selected marker compounds or the comparison of chemical fingerprints is widely used to define acceptable quality [1]. While these methods are indispensable for ensuring safety and authenticity, they are inherently limited in their ability to capture variation arising from epigenetically programmed metabolic states. If medicinal plants can exist in multiple stable regulatory configurations, then standardization based solely on fixed chemical criteria may be conceptually insufficient. Under such conditions, apparent variability in efficacy may reflect limitations of evaluative frameworks rather than intrinsic unpredictability of medicinal action.

This perspective calls for a redefinition of consistency in TCM. Instead of aiming for absolute chemical uniformity, it may be more appropriate to define therapeutically relevant ranges of metabolic states. Such an approach would acknowledge biological variability while still enabling reproducible outcomes. Incorporating information on cultivation history and regulatory context could enhance the interpretive power of quality assessment and align standardization more closely with biological reality.

### Toward epigenetically informed materia medica

**Fig. 1:**
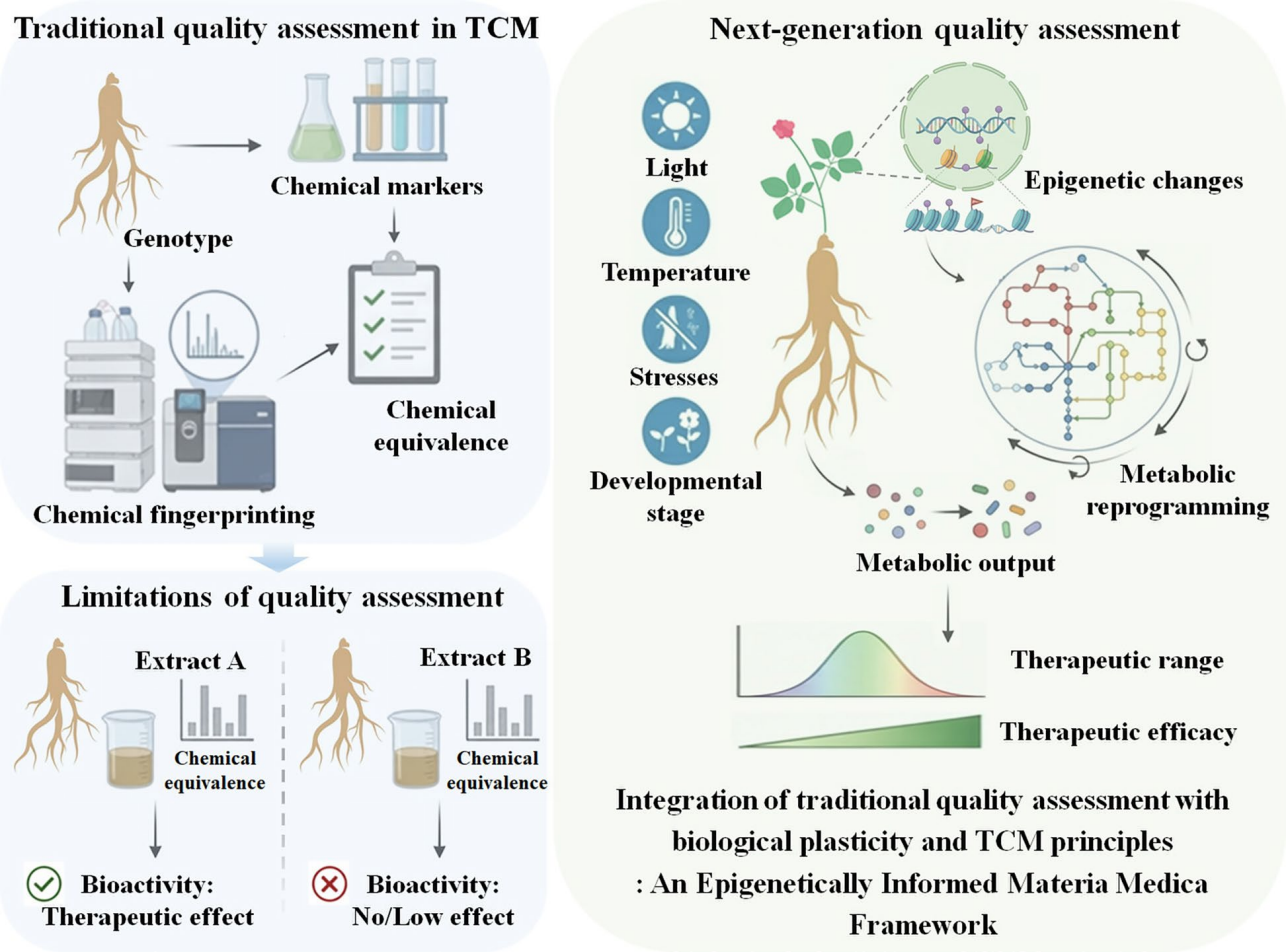
Conceptual framework illustrating why chemical equivalence does not reliably predict therapeutic equivalence in medicinal plants. Traditional quality assessment in traditional Chinese medicine (TCM) emphasizes chemical fingerprinting and marker-based equivalence, implicitly assuming that chemical composition alone is sufficient to ensure consistency. However, plants exhibit epigenetic and metabolic plasticity, whereby environmental and developmental factors establish stable regulatory states that reprogram metabolic networks. Consequently, extracts with similar chemical composition can display distinct bioactivity. An epigenetically informed materia medica framework integrates traditional chemical evaluation with dynamic metabolic organization and regulatory history to define therapeutically relevant functional ranges rather than composition-based chemical equivalence

An epigenetically informed materia medica framework can be proposed as a next-generation approach to quality assessment in TCM (Fig. 1). Operationally, this framework integrates metabolomic and epigenomic profiling to define regulatory states associated with consistent therapeutic performance. As illustrated by species such as *A. annua* and *G. uralensis*, functionally relevant variation often arises from coordinated shifts in metabolic networks shaped by environmental and regulatory history, rather than from isolated changes in individual compounds. Rather than relying exclusively on the absolute quantification of individual marker compounds, this framework emphasizes metabolic balance and pathway coordination as more informative indicators of biological function. Relative relationships among metabolites provide insight into the integrated activity of biosynthetic networks, which are shaped by the regulatory state of the plant. Such dynamic chemical signatures better capture how metabolic flux and pathway organization contribute to therapeutic potential than static concentration-based metrics.

In parallel, incorporating epigenetic features into quality evaluation offers a means to assess the regulatory history underlying metabolic behavior. In this framework, particular emphasis is placed on epigenetic features directly linked to metabolic regulation. DNA methylation at promoter regions of genes involved in secondary metabolism provides a tractable indicator of transcriptional potential, while histone modifications associated with active or repressed chromatin states reflect pathway-level regulatory activity. These features are prioritized because they directly regulate gene expression and enable coordinated control of entire metabolic pathways, rather than isolated enzymatic steps. As such, they provide a mechanistic link between regulatory configuration and the emergence of distinct metabolic states relevant to therapeutic function. These features are especially informative when they correlate with coordinated changes in metabolic networks, such as shifts in metabolite ratios or pathway flux, which are more closely aligned with functional outcomes than absolute metabolite abundance. In this context, epigenetic indicators serve not only as markers of regulatory history but also as predictors of metabolically and functionally distinct states relevant to therapeutic efficacy. Such signatures reflect how regulatory history, shaped by biological and environmental contexts, is translated into stable regulatory states that persist beyond the immediate growth environment. By linking epigenetic profiles with metabolic organization, this framework provides a mechanistic basis for understanding how cultivation history influences efficacy-relevant properties. This prioritization enhances practical feasibility, as these features can be measured with increasing reliability using current epigenomic approaches. In practice, this framework can be implemented by establishing reference profiles of metabolic network organization and associated epigenetic states from well-characterized, high-efficacy plant materials. Subsequent batches can then be evaluated based on their alignment with these defined regulatory states, rather than solely on absolute chemical composition. Together, integrating dynamic metabolic signatures with epigenetic indicators provides a biologically grounded basis for quality assessment that moves beyond static chemical equivalence. This approach enables the evaluation of medicinal plants as regulated biological systems and provides a foundation for predictive and reproducible standardization strategies that align with the complex nature of herbal therapeutics.

## Conclusion

Recognizing medicinal plants as epigenetically programmed systems offers a transformative direction for standardization in TCM. Rather than pursuing strict chemical uniformity, future frameworks should integrate dynamic metabolic signatures with epigenetic indicators to define therapeutically relevant ranges of functional states. Such a shift enables mechanistic connections between cultivation history, regulatory configuration, and therapeutic efficacy, thereby improving reproducibility across research and clinical practice. Ongoing advances in epigenomics, metabolomics, and systems pharmacology provide the technical foundation for developing regulation-compatible quality standards that accommodate biological plasticity while maintaining predictable outcomes. By aligning quality assessment with the systemic and adaptive principles underlying TCM, this approach lays the groundwork for a biologically realistic and functionally robust materia medica suitable for modern clinical and regulatory contexts.

## Data Availability

No datasets were generated or analysed during the current study.

## Abbreviations

TCM: Traditional Chinese Medicine
